# Supplementary material for: Appearance and suppression of Turing patterns under a periodically forced feed
Source: Commun Chem. 2023 Jan 3;6:3. doi: 10.1038/s42004-022-00800-6 (PMC9814632; doi:10.1038/s42004-022-00800-6)
Supplement: Supplementary file 2 — Supplementary Information [file 42004_2022_800_MOESM2_ESM.pdf]

Supplementary Information

Appearance and suppression of Turing patterns under a periodically forced feed

Brigitta Dúzs, Gábor Holló, Hiroyuki Kitahata, Elliott Ginder, Nobuhiko Suematsu, István Lagzi,* and István Szalai*

Supplementary Figures

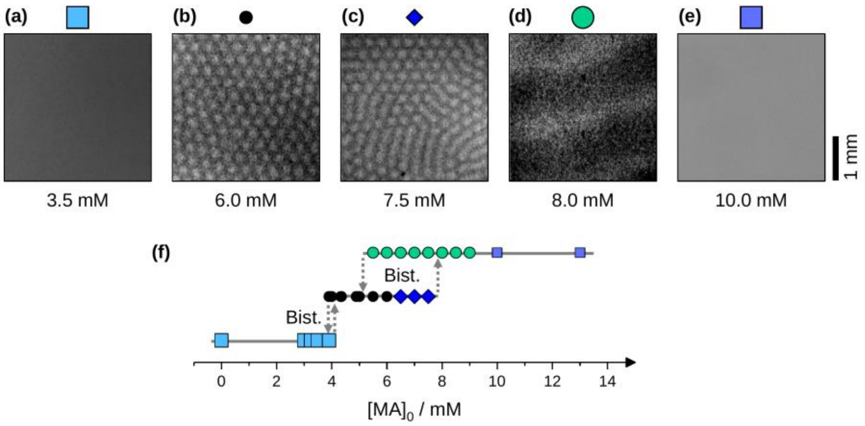

Supplementary Figure S1. Spatiotemporal behaviors as a function of constant boundary $[MA]_0$ concentration, without periodic forcing. Snapshots of the stabilized behaviors: no pattern (**a**), stationary Turing spots (**b**), mixed stationary Turing spots and stripes (**c**), waves (**d**), and no pattern (**e**). Concentration regimes of the dynamic behaviors (**f**): hysteresis was investigated by systematically increasing and then decreasing the $[MA]_0$ control parameter (bottom and top line of symbols in (**f**), respectively). Bistability, i.e., the overlap of the $[MA]_0$ range of different patterns was observed between the arrows. The residence time in each tank is 10.8 min. The screening experiments were repeated at least 2 times in different gels.

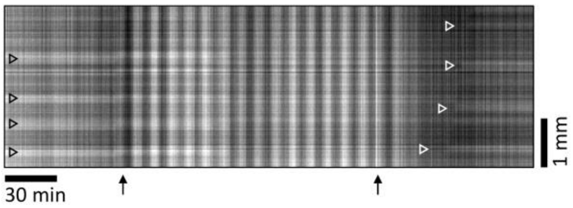

Supplementary Figure S2. Space-time plot of the temporary disappearance of stationary Turing spots induced by periodic forcing. The arrows indicate the start and end of the periodic forcing. The triangles show the stationary Turing spots (horizontal bands in the space-time plot). The vertical lines, i.e., time-periodic phenomena, appear due to the slight color change in tank B due to the periodic forcing. The corresponding experiment is presented in Figure 1b and Supplementary Movie 1.

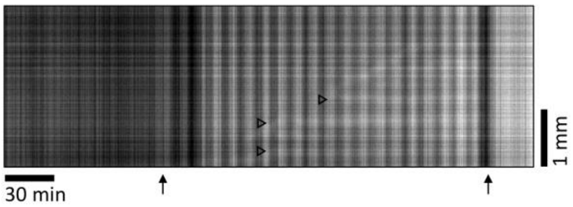

Supplementary Figure S3. Space-time plot of the temporary appearance of stationary Turing spots induced by periodic forcing. The arrows indicate the start and end of the periodic forcing. The triangles show the stationary Turing spots (horizontal bands in the space-time plot). The vertical lines, i.e., time-periodic phenomena, appear due to the slight color change in tank B due to the periodic forcing. The corresponding experiment is presented in Figure 1c and Supplementary Movie 2.

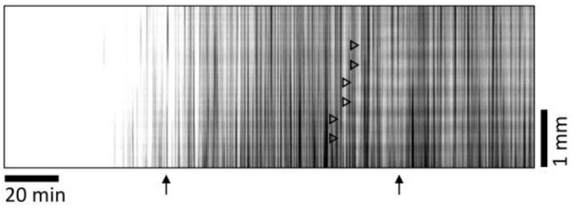

Supplementary Figure S4. Space-time plot of the appearance (and remaining) of stationary Turing spots induced by periodic forcing. The arrows indicate the start and end of the periodic forcing. The triangles show the stationary Turing spots (horizontal bands in the space-time plot). The corresponding experiment is presented in Figure 1d and Supplementary Movie 3.

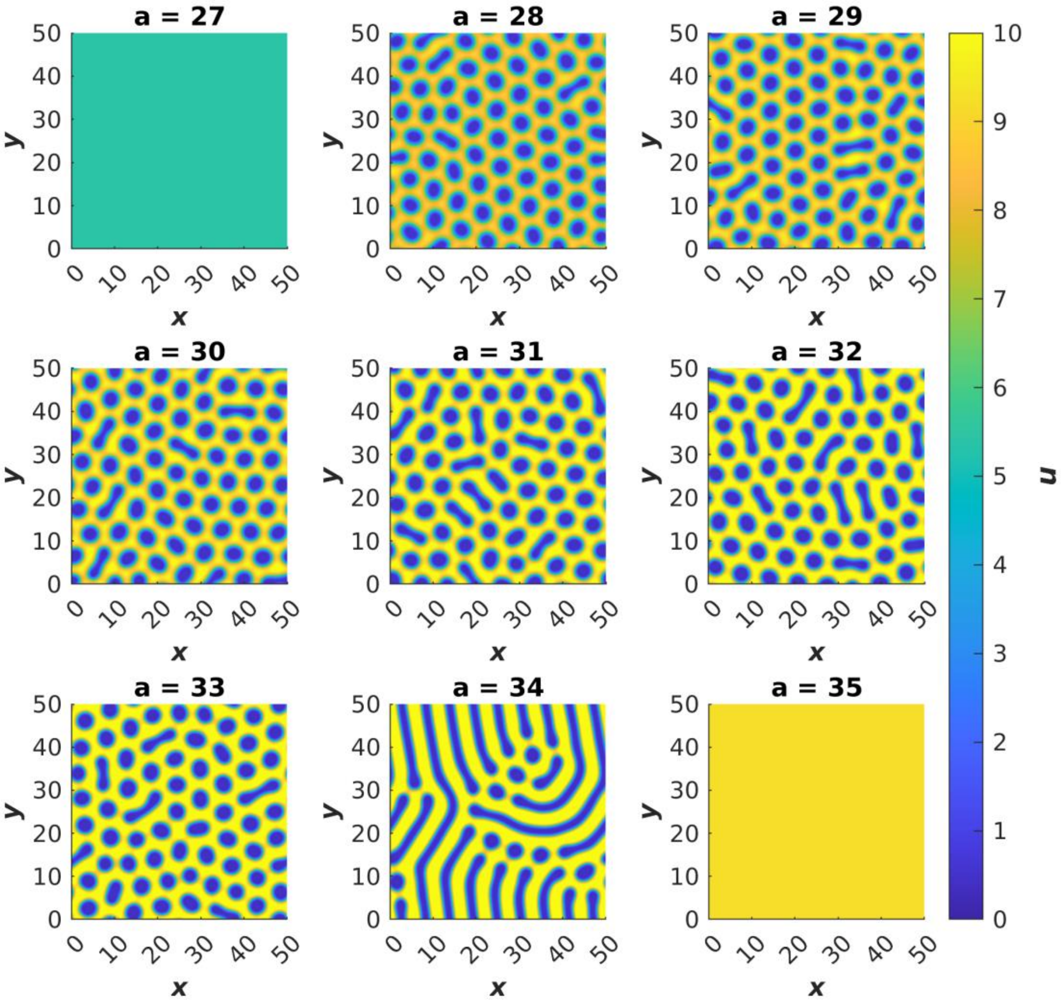

Supplementary Figure S5. Effect of the constant feed concentration (a) on the morphology of the pattern in numerical simulations. Below $a = 23$ and above $a = 27$ stationary homogeneous pattern and homogeneous oscillation exist, respectively. The following parameter set was used: $\sigma = 8$, $b = 1.9$, $c = 1.5$, $\Delta x = \Delta y = 1.25 \times 10^{-1}$ (grid spacing), and $\Delta t = 3.124 \times 10^{-4}$ (time step). The length of the simulation domain and the simulation time were 50×50 and 200, respectively. All parameters are dimensionless. The simulations were started from the same homogeneous initial state.

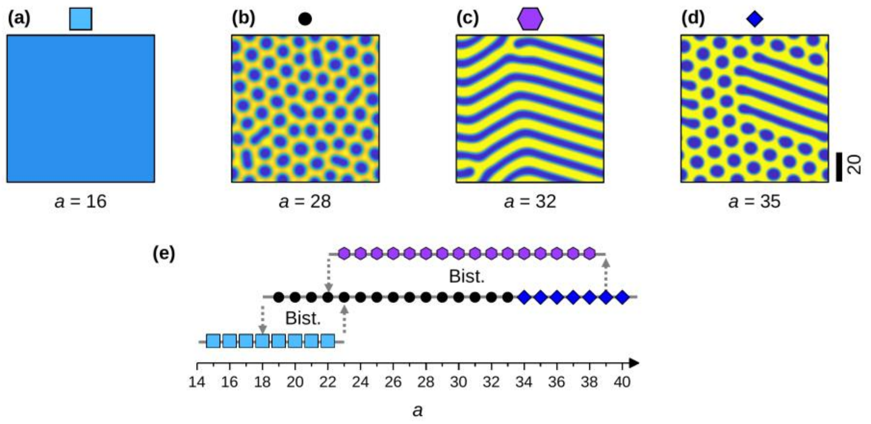

Supplementary Figure S6. Spatiotemporal behaviors as a function of constant feed concentration (a) in the numerical model, without periodic forcing. Snapshots of the stabilized behaviors: no pattern (**a**), stationary Turing spots (**b**), Turing stripes (**c**), and mixed Turing spots and stripes (**d**). Concentration regimes of the dynamic behaviors (**e**): hysteresis was investigated by systematically increasing and then decreasing the a control parameter. Two symbols at the same a indicate bistability (**e**). The following parameter set was used: $\sigma = 8$, $b = 1.9$, $c = 1.5$, $\Delta x = \Delta y = 2.5 \times 10^{-1}$ (grid spacing), and $\Delta t = 1.3 \times 10^{-3}$ (time step). The length of the simulation domain and the simulation time were 50×50 and 8000, respectively. The parameter a was increased from a = 15 to a = 40 every $t = 156.86$ and then decreased in the same manner. All parameters are dimensionless. In order to follow the stability of the different states, in each step the simulation have started from the final state of the previous step.

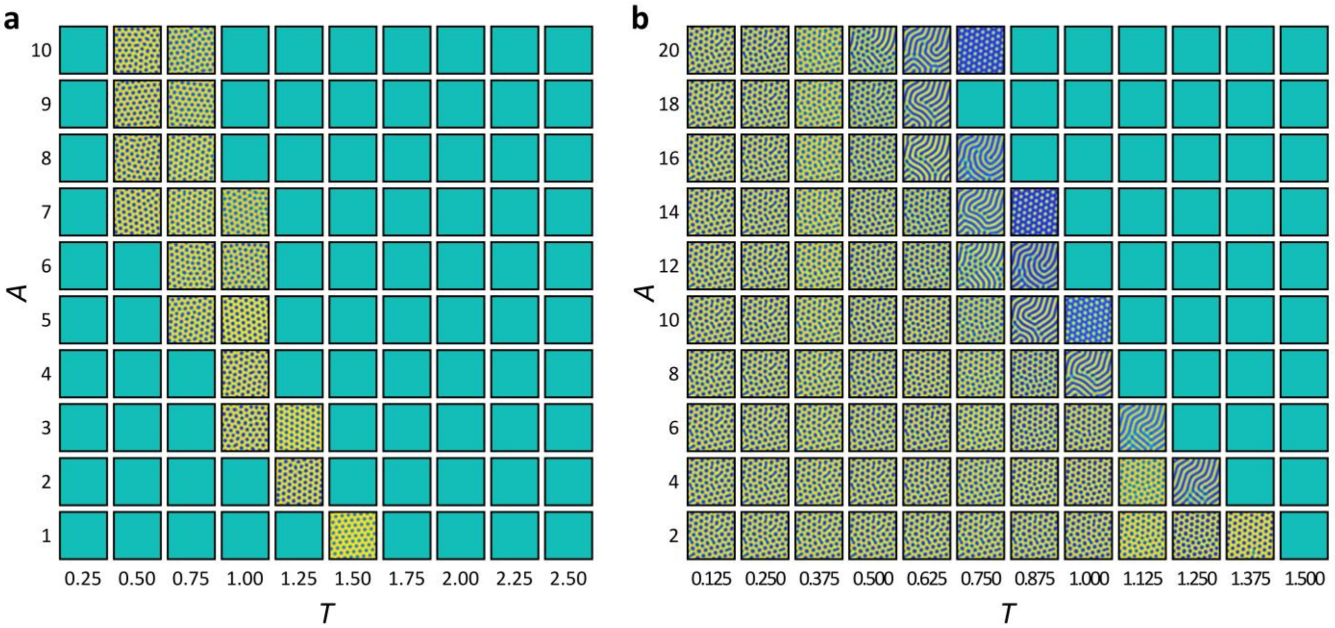

Supplementary Figure S7. Phase diagrams in the period-amplitude plane of the periodic forcing for two different initial feed concentrations (*a*). Each data point corresponds to independent simulations that were started from a homogeneous (**a**) or Turing (**b**) state. The Turing state indicates the appearance of any patterns (spotted, striped or their combination). The symbols/pictures show the behavior that stabilized during the periodic forcing: homogeneous bluish color indicates forcing-induced oscillation with the period of the applied forcing, the mosaic pictures of patterns indicate forcing-induced quasi-stationary behavior (Turing spots or stripes). The following parameter set was used: $\sigma = 8$, $b = 1.9$, $c = 1.5$, $\Delta x = \Delta y = 2.5 \times 10^{-1}$ (grid spacing), and $\Delta t = 6.25 \times 10^{-4}$ (time step). The length of the simulation domain and the simulation time were 50×50 and 400, respectively. All parameters are dimensionless.

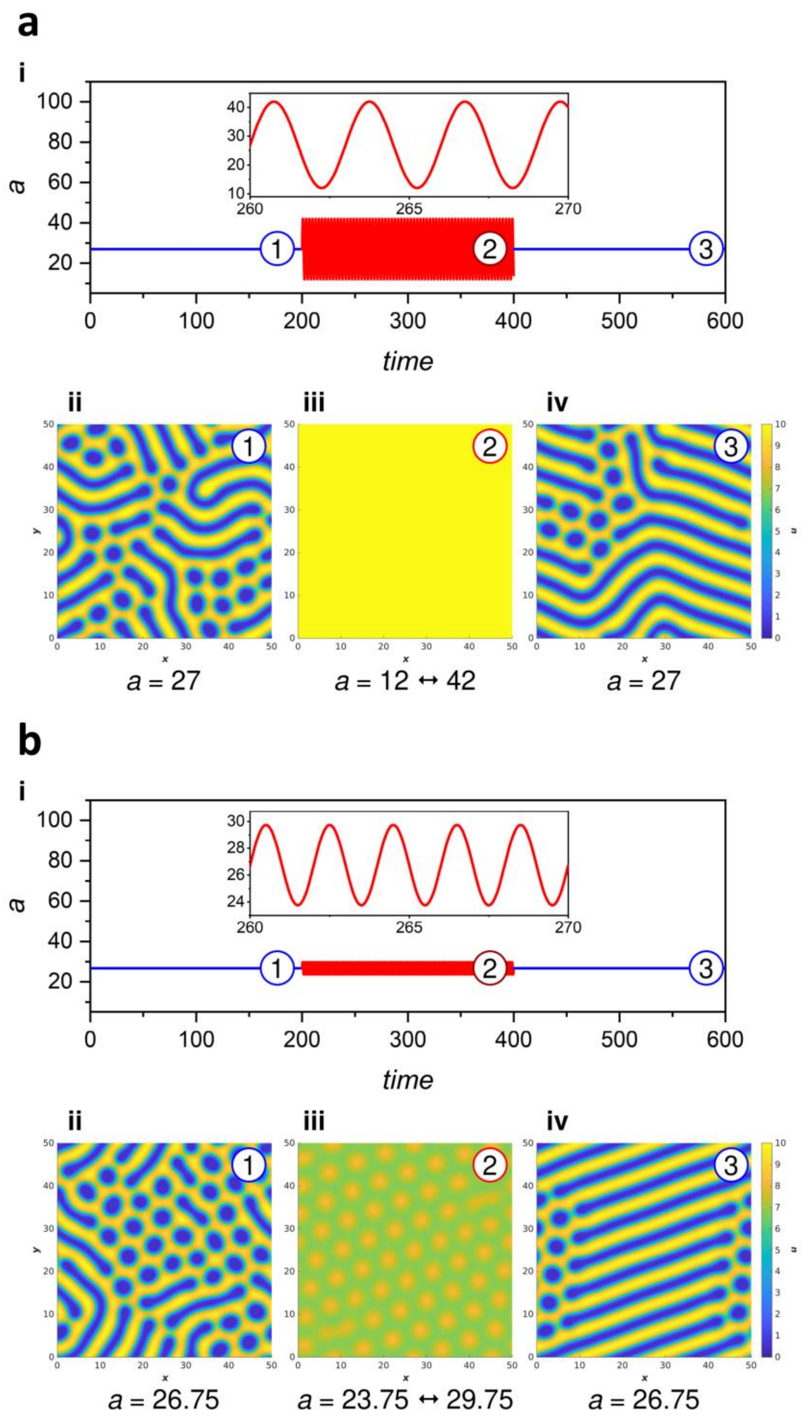

Supplementary Figure S8. Periodic forcing of the feed concentration (a) in numerical simulations. Disappearance of stationary mixed Turing spots and stripes induced by the periodic forcing of the feed concentration (a). The initial $a = 27$ was modulated with the amplitude of 15 and a time period of 3, respectively (**a**). The appearance of spots from stationary Turing stripes and spots induced by the periodic forcing of the feed concentration (a). The initial $a = 26.75$ was modulated with the amplitude of 3 and a time period of 2, respectively (**b**). In figures (**a**)-(**b**): (**i**) Change of the feed concentration (a) during the whole simulation. (**ii**)-(**iv**) Snapshots of the pattern generated with constant a before the forcing (1), periodic forcing of a (2), and constant a after the forcing (3). The following parameter set was used: $\sigma = 8$, $b = 1.9$, $c = 1.5$, $\Delta x = \Delta y = 1.25 \times 10^{-1}$ (grid spacing), and $\Delta t = 3.124 \times 10^{-4}$ (time step). The length of the simulation domain and the simulation time were 50×50 and 600, respectively. All parameters are dimensionless.

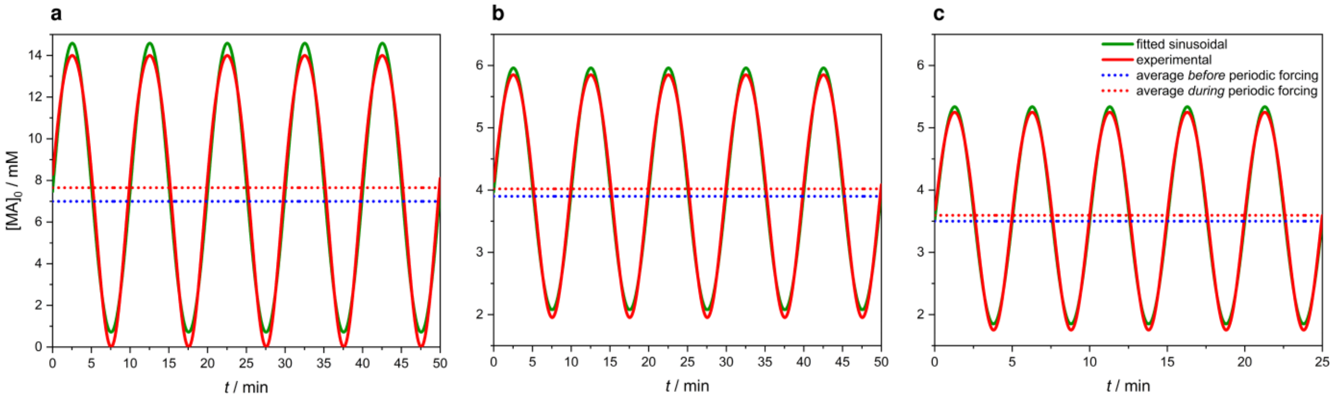

Supplementary Figure S9. Periodic modulation of the feed concentration of MA due to the sinusoidal modulation of the inflow rate in the experiments. Periodically modulated experimental $[MA]_0$ (red), fitted pure sinusoidal function (green) for different q_0 and A values (**a**-**c**). The red experimental curve was calculated using Equations (4)-(5) in the main text. The differential equation (5) was solved in MATLAB with the ode45 solver using relative tolerance: 10^{-8}, absolute tolerance: 10^{-10}. The time-independent $[MA]_{0,basis}$ before the periodic modulation (dotted blue) and the average of the periodically modulated $[MA]_0$ (dotted red). Figures (**a**), (**b**), and (**c**) correspond to experiments shown in Figure 2b, 2c, 2d in the main text, respectively.
