# Supplementary material for: Appearance and suppression of Turing patterns under a periodically forced feed
Source: Commun Chem. 2023 Jan 3;6:3. doi: 10.1038/s42004-022-00800-6 (PMC9814632; doi:10.1038/s42004-022-00800-6)
Supplement: Supplementary file 3 — Description of Additional Supplementary Files [file 42004_2022_800_MOESM3_ESM.pdf]

Description of Additional Supplementary Files

File name: Supplementary Movie 1

Description: The temporary disappearance of stationary Turing spots induced by periodic forcing in an experiment.

File name: Supplementary Movie 2

Description: The temporary appearance of stationary Turing spots induced by periodic forcing in an experiment.

File name: Supplementary Movie 3

Description: The appearance (and remaining) of stationary Turing spots induced by periodic forcing in an experiment.

File name: Supplementary Movie 4

Description: The temporary disappearance of stationary Turing spots induced by periodic forcing in a numerical simulation.

File name: Supplementary Movie 5

Description: The appearance (and remaining) of stationary Turing spots induced by periodic forcing in a numerical simulation.

File name: Supplementary Movie 6

Description: The temporary disappearance of stationary Turing stripes induced by periodic forcing in a numerical simulation.

File name: Supplementary Movie 7

Description: Transition from Turing stripes to spots induced by periodic forcing in a numerical simulation.
